# Proof‐of‐concept study of anti‐Fel d 1 IgY antibodies in cat food using the MASK‐air^®^ app

**DOI:** 10.1002/clt2.12353

**Published:** 2024-04-27

**Authors:** Jean Bousquet, Alina Gherasim, Frédéric de Blay, Eve Mathieu‐Dupas, Géraldine Batot, Daniel Laune, Bernardo Sousa‐Pinto, Torsten Zuberbier, Nhân Pham‐Thi, MASK‐cat study group, Bernard Hofmann, Emilie Urban‐Kraemer, Van‐Mai Nguyen‐Grosjean, Véronique Lustgarten, Catherine Defrance

**Affiliations:** ^1^ Institute of Allergology Charité—Universitätsmedizin Berlin Corporate Member of Freie Universität Berlin and Humboldt‐Universität zu Berlin Berlin Germany; ^2^ Fraunhofer Institute for Translational Medicine and Pharmacology ITMP, Immunology and Allergology Berlin Germany; ^3^ MASK‐air Montpellier France; ^4^ ALYATEC Nouvel hôpital Civil‐Hôpitaux Universitaires Stransbourg France; ^5^ Allergy Division, Chest Disease Department University Hospital of Strasbourg Strasbourg France; ^6^ Federation of Translational Medicine University of Strasbourg Strasbourg France; ^7^ KYomed INNOV Montpellier France; ^8^ MEDCIDS—Department of Community Medicine, Information and Health Decision Sciences, Faculty of Medicine, University of Porto Porto Portugal; ^9^ CINTESIS@RISE ‐ Health Research Network, Faculty of Medicine, University of Porto Porto Portugal; ^10^ Ecole Polytechnique de Palaiseau Palaiseau France; ^11^ IRBA (Institut de Recherche Bio‐Médicale des Armées) Brétigny sur Orge France; ^12^ Université Paris Cité Paris France

**Keywords:** anti‐Fel d 1 antibodies, asthma, cat allergy, cat food, combined symptom‐medication score, conjunctivitis, rhinitis

## Abstract

**Background:**

An innovation to better manage cat‐allergic patients utilises anti‐Fel d 1 IgY antibodies to neutralise Fel d 1 after its production by the cat. However, there is no published study showing its clinical efficacy in humans in a home setting. A longitudinal, open‐label, proof‐of‐concept study was carried out to approach clinical efficacy of the cat food in cat‐allergic patients.

**Methods:**

After a baseline evaluation, the cats ate only the cat food for the following 4 months. Daily evaluation of efficacy was performed for 2 weeks at baseline and after 1, 2 and 3 months of intervention for periods of 2 weeks. The MASK‐air app was used daily to assess symptoms, work productivity and medications.

**Results:**

Of the 49 patients screened, 42 were followed up and 33 (78.5%) reported MASK‐air data at all 3 evaluation periods. The primary end point (visual analogue scale [VAS] for global allergy symptoms) was significantly improved (*p* < 0.0001). All symptoms (VAS nose, eye, and asthma), VAS work and the combined symptom‐medication score significantly improved after 1 month. The percentage of uncontrolled days (VAS>20/100) decreased from 64% at baseline to 35% at 1 month (*p* < 0.0001) and 14% at 3 months. A sensitivity analysis in patients with uncontrolled disease at baseline found similar results.

**Discussion:**

A cat diet containing anti‐Fel d 1 antibodies was able to (i) show decreased allergic symptoms and related outcomes, (ii) inform the design and feasibility of future studies with a control arm and (iii) estimate the sample size of the study.

**Study registration number**: clinicaltrials.gov: **NCT05656482**.

## 1 INTRODUCTION

The prevalence of allergy to furry animals has been increasing. Allergy to cats is a major risk factor for the development of asthma and rhinitis.^1^ The human‐animal bond is strong in owners allergic to cats^2^ and many allergic patients try to keep their cat despite experiencing symptoms. Allergen avoidance is effective^1^ but often has a psychological impact. Allergen immunotherapy is not well demonstrated.^3^ There is a need for innovative approaches to better manage cat allergy. Biologics may be a future approach, but their cost‐effectiveness is unknown.^4^, ^5^, ^6^

An innovation to better manage cat‐allergic patients utilised anti‐Fel d 1 IgY antibodies to safely and effectively neutralise Fel d 1 after its production by the cat. The efficacy of a feline diet with an egg‐product ingredient containing anti‐Fel d 1 IgY antibodies was demonstrated in vitro, ex vivo and in vivo, and a pilot exposure study involving cat‐allergic human participants suggested its efficacy.^7^, ^8^

MASK‐air^®^ (Mobile Airways Sentinel networK) is a mobile health app that assesses the daily control of allergic rhinitis and asthma.^9^ It has been freely available since 2015 on the Apple App and Google Play Stores and is currently available in 28 countries. MASK‐air has been classified as a Good Practice of DG Santé for digitally enabled, patient‐centred care in rhinitis and asthma multimorbidity.^10^ It is one of the 13 Best Practices of OECD (Organisation of Economic Cooperation and Development) in integrated care for chronic diseases.^11^ It is registered as a medical device regulation Class IIa and fully complies with the General Data Protection Regulation.

We aimed to estimate the efficacy of the cat food on symptoms, medications and work in cat‐allergic patients enrolled by allergists. This was a proof‐of‐concept study as the MASK‐air^®^ app has not previously been used to measure the correlation between allergic symptoms and a cat food within a home environment. Furthermore, there was no published clinical study on the cat food. However, it was recognised that several confounding variables may affect the feasibility of demonstrating a significant correlation between the cat food and symptoms using this approach.

## 2 METHODS

### 2.1 Design

Using MASK‐air^®^, a longitudinal, open label, proof‐of‐concept study was carried out to assess whether a cat diet containing anti‐Fel d 1 antibodies was able to (i) decrease allergic symptoms and related outcomes, (ii) inform the design and feasibility of future studies with a control arm and (iii) estimate the sample size. We used primary (visual analogue scale [VAS] global allergy symptoms assessed using the MASK‐air^®^ app between baseline and 3 months) and additional secondary (Combined symptom‐medication score [CSMS], VAS rhinitis, eye, asthma and work as well as the percentage of symptom‐free days) end points between baseline and 3 months. Moreover, we compared data at baseline, Days 42–56, Days 70–84 and Days 98–112.

### 2.2 Settings

Patients were included in outpatient clinics around France from May 2022 to April 2023 (Table S1 online).

### 2.3 Patients

Patients of both sexes ranging in age from 18 to 70 years and with current worsening of symptoms when exposed to one or two cats living in the household were enrolled. Cats had to have a diet consisting mainly of dry kibble. Patients agreed to change their cat(s’)'s food and feed it/them exclusively with the test kibble during the study. Cat sensitisation was assessed by a positive prick test (greater than 5 mm more than the negative control and a positive histamine control) to standardised cat allergens (Stallergènes‐Greer or ALK) or serum cat‐specific IgE. The cat sensitisation reported by the physician should have been assessed within the past 5 years. The physician also carefully reported sensitisation to other allergens and symptoms during pollen season to avoid including the patient during the exposed season. The patient should not have been sensitised to pollen during the study period. Any medication except oral corticosteroids was allowed. The patient should not have had cat immunotherapy.

The exclusion criteria are listed in Table S2 online.

### 2.4 Cat food

The test diet was a commercially available dry feline diet (Purina Pro^®^ Plan LiveClear^TM^) formulated and manufactured by Nestlé Purina PetCare Company (St. Louis, Missouri, USA). Previously published studies documented the diet's safety for cats^12^ as well as its efficacy in reducing Fel d 1 in cats' saliva^7^ and hair.^13^

### 2.5 Outcomes

MASK‐air^®^ includes a daily monitoring questionnaire assessing the impact of allergy symptoms through four mandatory VASs on a 0 to 100 scale (Table S3 online). When reporting daily VAS, MASK‐air^®^ users provide their daily medication use through a scroll list customised for each country and regularly updated.^9^ We also studied VASs on work productivity.^14^

Symptom and medication data daily provided by patients allow the calculation of the daily combined symptom‐medication scores from formulae previously published (CSMS).^15^

Cut‐off values for “controlled”, “partly controlled” and “uncontrolled” outcomes have been previously determined.^16^

The primary end point was a significant reduction of the “overall” VAS between “baseline” and Days 98–112. Hierarchical secondary end points were defined (Table S4 online).

### 2.6 Study protocol

The patients' cat(s’)'s current feeding regimen diet was to be primarily around 40–80 g per day of dry kibble. Patients accepted to transition their cat(s’)'s food and to feed it/them exclusively on dry kibble for the duration of the trial. Patients were instructed to maintain all usual medications, cleaning and other practices throughout the study so that the only change made would be their cat(s’)'s diet.

**FIGURE 1:**
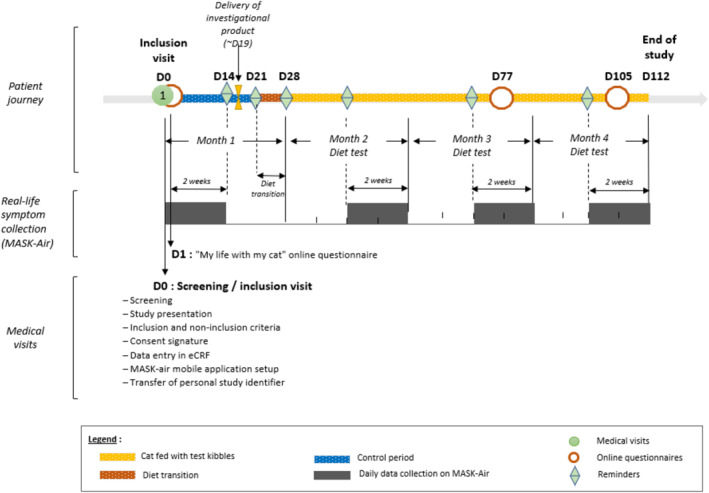
Study protocol.

The study protocol is shown in Figure 1 and Tables S5 and S6 online. The protocol was validated by ARIA (Allergic Rhinitis and its Impact on Asthma) members (Bernardo Sousa Pinto, Torsten Zuberbier). Each patient was followed over the 112‐day period during which the cat(s) ate only the test kibble, with 2‐week assessments at baseline and from Days 42 to 112.

A food transition period was observed between Days 14 and 28, according to instructions provided by the Physician on Day 0 (visit#1, Table S6 online).

This was a partially dematerialised study in which data were collected from volunteers using the MASK‐air^®^ digital tool. Completion of the questionnaires daily was entirely voluntary. No medical visit was carried out after the inclusion of the patient.

Patients filled in the MASK‐air^®^ app during four study periods: Days 0–14 (baseline), Days 42–56 (P1), Days 70–84 (P2) and Days 98–112 (P3).

### 2.7 Sample size

As this was a pilot study, the sample size was not pre‐calculated. However, based on estimations, we determined that a minimum of 40 participants was necessary for the study. Taking into account potential loss of follow‐up and dropouts during the study, which was estimated at 10%, the final number of subjects to be recruited was approximately 50.

### 2.8 Statistical analysis

Categorical variables were described using absolute frequencies and percentages. Demographic continuous variables were described using means and standard deviations. The remaining continuous variables were non‐Gaussian and were described using medians and percentiles.

We applied mixed‐effect linear regression models having reported VAS or CSMS levels as the dependent variable and the study period as the independent variable, with the identification of the participant being set as the random effect (i.e., we clustered observations by participants). Therefore, we were able to obtain regression coefficients for the differences in VAS/CSMS levels in the comparison between each subsequent period and baseline.

We applied the chi‐square test for the comparison across periods on (i) the frequency of patients with partly or poorly controlled rhinitis (i.e., patients with a median VAS global ≥20 at baseline) and (ii) the frequency of days with partly or poorly controlled rhinitis (i.e., VAS global ≥20).

No imputation of missing data was performed.

## 3 RESULTS

### 3.1 Demographic characteristics of the patients

A total of 49 patients provided their consent for the study. However, 5 of these patients did not enter their data in the MASK‐air^®^ app and were excluded from the analysis. Thus, 45 patients successfully completed the MASK‐air^®^ daily monitoring questionnaire at least once during the baseline period, and 29 completed all the study period assessments (65.9%). Moreover, 36 patients completed P1, 38 P2 and 29 P3 (Table 1 and Figure S1 online).

**TABLE 1:** Demographic characteristics of the patients.

Characteristic	
Total number of enrolled patients	49
Age (years), mean (SD)	37.5 (10.3)
Female, *n* (%)	35 (71%)
Rhinitis, *n* (%)	49 (100%)
Asthma, *n* (%)	26 (53%)
Allergy, *n* (%)	
Other animals	12 (24%)
Dust mite	20 (41%)
Mould	2 (4%)
Cockroach	1 (2%)
Pollens	24 (49%)
Birch pollen	14 (29%)
Grass pollen	14 (29%)
Cypress pollen	5 (10%)
*Parietaria* pollen	0 (0%)
Olive pollen	9 (18%)
Ragweed	0 (0%)
Other trees	1 (2%)

Overall, the study diet was well tolerated except for two cats. Two patients dropped out of the study, one because their cat did not tolerate the food and the other because their cat gained a lot of weight.

All patients had rhinitis and more than half (*n* = 26; 53%) had asthma. Patients ranged in age from 20 to 61 years, and most were sensitised to other allergens, mainly pollens (Table 1).

Of the 44 individuals who provided information on the characteristics of their cat(s), 15 subjects had two cats. The characteristics of the first and second cats are given in Table S7 online. Overall, the cats were mostly short‐haired, sterilised and living indoors.

Of the 44 individuals who provided information on their quality of life with their cat(s), Table S8 online indicates the characteristics according to 7‐point Likert scales: from 1 (totally disagree) to 7 (completely agree). Mean values ranged from 4 ± 1.9 to 4.4 ± 2.5.

### 3.2 Adherence to the app

Overall, participants provided a total number of 1421 days of MASK‐air^®^ use during the study period. This corresponds to an overall app adherence of 52.6%. The mean number of days of MASK‐air^®^ use of the app ranged from 11.3 (baseline) to 7.3 (P3) (Table 2 and Figure S2 online).

**TABLE 2:** Number of days reported.

	Mean	min	Q1	Q2	Q3	Max
Over the study period	46.8	1	29	40	58	124
Number of uses	Mean	min	Q1	Q2	Q3	max
Baseline period	11.3	1	10	13	15	21
P1	9.6	1	8	10	13	16
P2	9.2	1	6.2	10	12	17
P3	7.3	1	3	8	11	14

At baseline, 32 patients used the app for over 10 days. This compares to 20 patients at P1, 21 at P2 and 10 at P3 (Figure S3 online).

### 3.3 Primary end point results in the entire study group and the 25 patients with four evaluations

**FIGURE 2:**
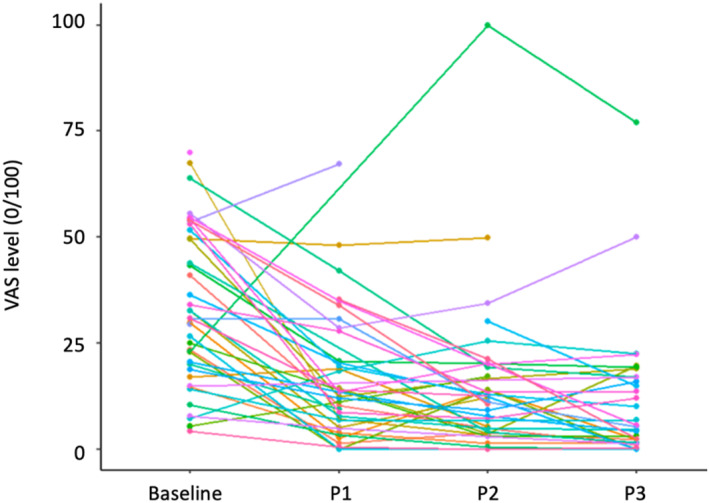
Global results in the 25 patients with all three evaluations.

**FIGURE 3:**
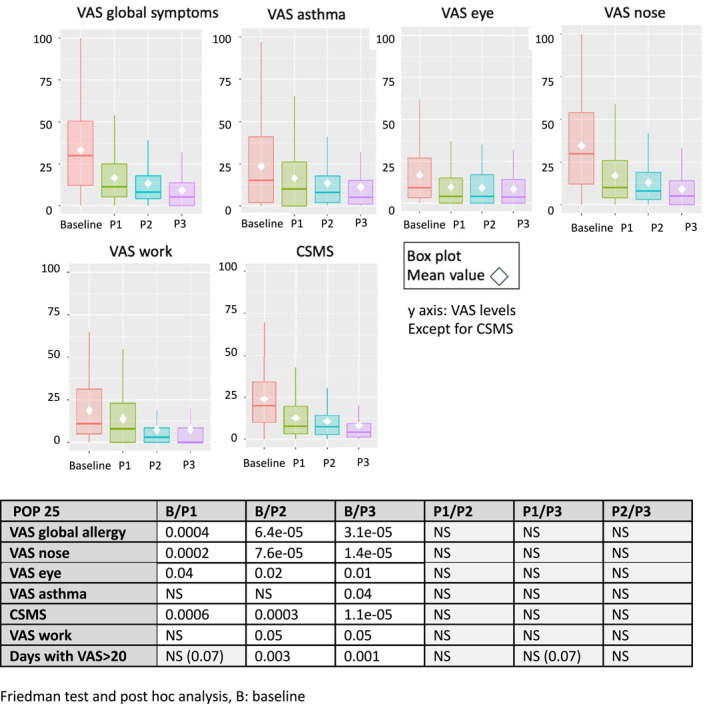
Median and mean results in the 25 patients with all three evaluations.

At baseline, there were 7 patients with median VAS global allergy symptoms <20/100 (controlled patient), 8 between 21 and 36/100 (partly controlled) and 14 > 36/100 (uncontrolled). There was a highly significant correlation between baseline and P3 (*p* < 0001). At 1 month, all but 5 patients had an improved VAS. The median levels were non‐significantly reduced between P1, P2 and P3 (Figures 2 and 3).

**FIGURE 4:**
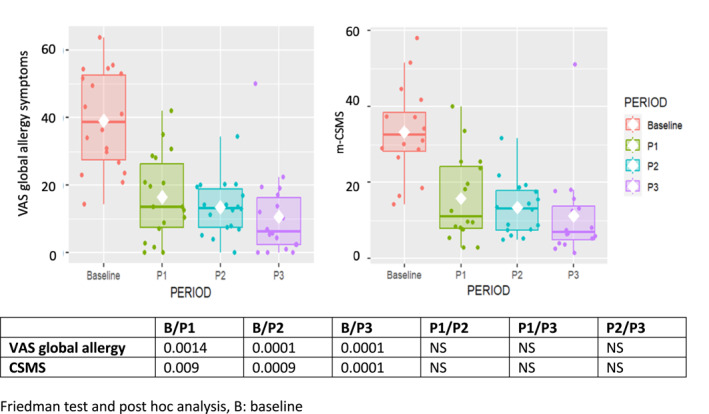
VAS global allergy symptoms and CSMS in 18 patients with uncontrolled symptoms at baseline and in all three evaluations. Results in medians and percentiles. CSMS, combined symptom‐medication score; VAS, visual analogue scale.

Consistent results were observed when solely assessing the 25 patients who provided MASK‐air^®^ data for all evaluation periods (Figure 4).

### 3.4 Secondary end point results in 25 patients with four evaluations

All other VASs and the CSMS were found to be significantly lower at P3 compared to the baseline period (*p* < 0.01 to *p* < 0.001, Wilcoxon W test) (Table 3). Significant results were also observed for all these outcomes when comparing values at P1 versus those at baseline (Figure S4 online).

**TABLE 3:** Percentages of controlled and uncontrolled days.

Percentage of days (size)	VAS	Baseline	P1	P2	P3	*p* value^a^		
All patients (%)						B/P1	B/P2	B/P3
*N*		415	318	309	219	2^e^‐16	2^e^‐16	2^e^‐16
Well controlled	<20	36% (148)	65% (206)	77% (238)	86% (*n* = 189)			
Moderately controlled	20–35.9	21% (87)	22% (71)	16% (50)	9% (*n* = 20)			
Uncontrolled	≥36	43% (180)	13% (41)	7% (21)	5% (*n* = 10)			
POP 25 (%)								
*n*		283	221	211	179	0.0006	0.0004	9^e^‐05
Well controlled	<20	40.6% (115)	67.4% (149)	77.7% (164)	86% (154)			
Moderately controlled	20–35.9	21.2% (60)	23.5% (52)	18% (38)	9.5% (17)			
Uncontrolled	≥36	38.2% (108)	9% (20)	4.3% (9)	4.5% (8)			

*Note*: Cutoff values used: VAS global<20: Well controlled, VAS global 20–35.9: Moderately controlled, VAS global≥36: Uncontrolled.

^a^ Chi‐squared test of comparison.

Although few patients reported asthma, VAS asthma showed a trend similar to other outcomes and there was a significant improvement at P3.

### 3.5 Days with poorly controlled or uncontrolled days

At baseline, for all patients, 36% of days were well controlled (VAS≥20/100) and 43% were uncontrolled (VAS>36/100). Well‐controlled days increased to 65% (P1), 77% (P2) and 86% (P3) (Table 3). Similar results were found in the selected population of 25 patients.

### 3.6 Sensitivity analysis in patients with uncontrolled symptoms (VAS global ≥20/100) at baseline

VAS global allergy symptoms and CSMS were studied in patients with median partly or uncontrolled‐days at baseline (Figure 4) and the same trend was observed. In particular, at P3, the levels of VAS or CSMS were similar in the entire as well as in selected populations.

## 4 DISCUSSION

The introduction of cat food containing anti‐Fel d 1 IgY antibodies was overall associated with an improvement in the primary outcome (global allergy symptoms at 3 months). In particular, there was a decrease in symptom severity, with less than 15% of the patients displaying partly or poorly controlled rhinitis symptoms at the end of the follow‐up period compared to more than two‐thirds at baseline. Moreover, there was a significant improvement in all outcomes after 1 month, possibly reflecting the kinetics of Fel d 1 in cats' saliva.

### 4.1 Limitations and strengths

The study has several limitations that should be considered carefully. First, there was no control group and the study can only be used as a proof‐of‐concept. It is clear that the best study should have been a placebo‐controlled study.^17^ However, to do so, the efficacy should be estimated and the sample size established. This was the reason for proposing a non‐controlled study. We have now established the sample size of a placebo‐controlled study (200 patients), the parameters to be studied and the study duration, with the primary end point at one month and not at 3 months. Thus, although the results are striking, they should be interpreted with care. In particular, it is not possible to exclude that the use of the MASK‐air^®^ app itself may have contributed to better rhinitis self‐care and symptom control. However, the magnitude of the response and the consistent effects across periods and tools suggest a true effect. Second, not all participants provided the same amount of MASK‐air^®^ data (with some participants not providing information on complete periods). While results are similar when considering data from all participants and data provided only by participants for all study assessment periods, we may not exclude the possibility that perceived lack of efficacy could be one of the reasons for not providing data.

Fel d 1 in saliva was not studied since it was not felt to be a major end point for a non‐controlled study and it would have made the study more complex.

The pollen season was excluded in patients allergic to pollen. However, some were also allergic to house dust mites, and we did not make a stratification of these patients due to limitations in sample size. Nevertheless, it is not plausible that changes in reported symptoms are mostly driven by changes in house dust mite exposure. In France, the other allergens (e.g. mould or cockroach) are not common sensitisers.

Finally, there might be differences depending on cat breeds, but this could not be tested in the current study. Female cats produce a lower level of allergens than males, and neutered males produce a lower level of allergens than full tom cats.^18^ In the present study, most cats were females and most males were neutered. The study should be confirmed in full tom male cats.

The study also has strengths. First, the patients were enrolled by allergists after a precise diagnosis of allergy to cats. Second, there was no stratification of patients depending on their control at baseline. Third, the app used is a Best Practice of the Directorate General Health and Food Safety (EU Commission) and of OECD (Organisation of Economic Cooperation and Development). It has been validated largely and used in many studies in rhinitis and asthma.^15^, ^19^ Fourth, one of the strengths of the MASK‐air app is to include all medications for allergic rhinitis and asthma. These medications are reported daily, allowing a careful monitoring of medications. We instructed the patients to maintain their medications; however, when they feel better, they often reduce them. The CSMS includes symptoms and medications. It was significantly reduced during the trial, indicating that patients reduced their medication needs. The study showed that the cat diet reduced patients' symptoms as well as their medications.

### 4.2 Interpretation of the data

There was an improvement in all symptoms at the first evaluation after baseline, as well as in the percentage of uncontrolled days. There was then a very low level for all outcomes and no significant differences between P1, P2 and P3. This rapid onset of clinical parameters accords with the levels of Fel d 1 in the saliva of treated cats,^7^ suggesting that the kinetics of the clinical findings may be associated with a reduction of Fel d 1 in cats. Interestingly, all outcomes (symptoms, CSMS, work productivity) were similarly improved. The median VAS values were rather low (25 (10; 48)), but this was due to patients with median baseline VAS levels of under 20/100. This improvement was also found in the subgroup of patients with uncontrolled disease at baseline (median VAS: 39/100).

An important finding was the percentage of uncontrolled days (VAS ≥20/100) at baseline (55%), P1,P2, and P3 (36%, 13% and 14%). These findings support the rapid effect of the cat food.

One improved domain was the impact on work productivity. In MASK‐air, VAS work has always been strongly associated with symptoms and with the CSMS.^9^, ^14^, ^20^, ^21^ Similar findings were found in this study. Since patients do not necessarily relate symptoms and work, these results strengthen the value of ours. Moreover, costs can be extrapolated from work productivity.

Following this study, a controlled study to confirm these results may be conducted. This proof‐of‐concept study may help to stratify patients since only uncontrolled patients with median VAS global allergy symptoms ≥20/100 should be enrolled in the new study. The number of patients to be included was defined according to this study. Moreover, although the primary end point should still be “VAS global allergy symptoms”, patients with asthma should be included. This warrants a separate analysis to compare the benefits on rhinitis and asthma. Finally, the economic value of the study should be considered as MASK‐air^®^ allows this evaluation using VAS work productivity,^9^, ^14^, ^21^ EQ‐5D^19^, ^22^, ^23^, ^24^ and the Work Productivity and Activity Impairment Allergic Specific.^15^, ^19^, ^25^ It is crucial that the design of a future study should include (i) the quantification of Fel d 1 levels in saliva and dander and (ii) the determination of sIgE in enrolled patients to all available single cat allergens.

The results of a POC study cannot be generalisable.

## 5 CONCLUSIONS

In this open‐label study, results can only be hypothesis‐generating and should be confirmed in properly designed controlled trials. However, the results have largely met the hypotheses raised. A cat diet containing anti‐Fel d 1 antibodies was able to (i) strongly decrease all allergic symptoms and reduce the impact of allergy symptoms on work and (ii) inform the design and feasibility of future studies with a control arm, including the estimation of the sample size. Moreover, the results were in line with the biological effect of the cat food on Fel d 1 saliva levels, with an improvement in symptom levels being observed after 1 month of follow‐up.

## AUTHOR CONTRIBUTIONS

Jean Bousquet proposed the study to Purina, wrote the protocol, analysed the data and wrote the paper. Alina Gherasim, Frédéric de Blay and Nhân Pham‐Thi reviewed the protocol, included the largest number of patients, analysed the data and reviewed the paper. Eve Mathieu‐Dupas, Géraldine Batot and Daniel Laune finalised the protocol, were the CRO of the study and performed the analyses. Bernardo Sousa‐Pinto and Torsten Zuberbier were the ARIA members reviewing the protocol and the results. All authors have reviewed and approved the manuscript.

## CONFLICT OF INTEREST STATEMENT

JB reports personal fees from Cipla, Menarini, Mylan, Novartis, Purina, Sanofi‐Aventis, Teva, Noucor, other from KYomed‐Innov, other from Mask‐air‐SAS, outside the submitted work. TZ reports grants and personal fees from Novartis, grants and personal fees from Henkel, personal fees from Bayer, personal fees from FAES, personal fees from Astra Zeneca, personal fees from AbbVie, personal fees from ALK, personal fees from Almirall, personal fees from Astellas, personal fees from Bayer, personal fees from Bencard, personal fees from Berlin Chemie, personal fees from FAES, personal fees from Hal, personal fees from Leti, personal fees from Mesa, personal fees from Menarini, personal fees from Merck, personal fees from MSD, personal fees from Novartis, personal fees from Pfizer, personal fees from Sanofi, personal fees from Stallergenes, personal fees from Takeda, personal fees from Teva, personal fees from UCB, personal fees from Henkel, personal fees from Kryolan, personal fees from L'Oreal, outside the submitted work; and Organisational affiliations: Commitee member: WHO‐Initiative "Allergic Rhinitis and Its Impact on Asthma” (ARIA); Member of the Board: German Society for Allergy and Clinical Immunology (DGAKI); Head: European Centre for Allergy Research Foundation (ECARF); President: Global Allergy and Asthma European Network (GA2LEN); Member: Committee on Allergy Diagnosis and Molecular Allergology, World Allergy Organisation (WAO). FdB reports other from NOVARTIS, other from ALK, other from STALLERGENES, other from REGENERON, other from DBV, other from SANOFI, other from BOEHRINGER, and other from ASTRAZENECA, outside the submitted work. The other authors have nothing to disclose, outside the submitted work.

## MASK‐CAT STUDY GROUP

- Bernard Hofmann, Carpentras, France
- Emilie Urban‐Kraemer, Fréjus, France
- Van‐Mai Nguyen‐Grosjean, Metz, France
- Véronique Lustgarten, Nice, France
- Catherine Defrance, Cagnes‐sur‐mer, France

## Supporting information

Supporting Information S1

## Data Availability

Data are not available.
