# Supplementary material for: Proof‐of‐concept study of anti‐Fel d 1 IgY antibodies in cat food using the MASK‐air® app
Source: Clin Transl Allergy. 2024 Apr 27;14(5):e12353. doi: 10.1002/clt2.12353 (PMC11055507; doi:10.1002/clt2.12353)
Supplement: Supplementary file 1 — Supporting Information S1 [file CLT2-14-e12353-s001.docx]

**Table S1 online: settings and number of patients enrolled**

| **Centre** | **Number of inclusions** |
| --- | --- |
| CAGNES-SUR-MER | 4 |
| CARPENTRAS | 2 |
| FREJUS | 4 |
| METZ | 2 |
| NICE | 5 |
| PARIS | 16 |
| STRASBOURG | 16 |

**Table S2 online: Exclusion criteria**

- Person with dogs or more than 2 cats
- Person planning to be away from the cat(s) for more than one week/month during the 4 months following the inclusion visit
- Patient who has had a new cat in the home for less than a month
- Patient in regular contact with other animals to which he/she is allergic
- Patient with uncontrolled asthma
- Patient receiving immunotherapy for cat allergies
- Patient participating or having participated in an allergic rhinitis study within the last 30 days or 5 half-lives of the study drug
- Person with difficulty understanding or reading the information note
- Person declaring to be under guardianship, curatorship or safeguard of justice.

**Table S3 online: Visual analogue scales (VASs) used in MASK-air^®^**

| **MASK-air^®^ VAS** | **Question** |
| --- | --- |
| VAS global allergy symptoms | Overall, how much are your allergic symptoms bothering you today? |
| VAS nose | How much are your nose symptoms bothering you today? |
| VAS eyes | How much are your eye symptoms bothering you today? |
| VAS asthma | How much are your asthma symptoms bothering you today? |

**Table S4 online: Primary and secondary end points**

**Primary Endpoint:**

• Significant reduction of the “overall” VAS between “baseline” and D98-D112.

The approach will be considered beneficial if there is a statistically significant decrease in this criterion over the period D98-D112 compared to the baseline.

**Hierarchical secondary endpoints:**

• Evolution of the “overall” VAS during the 4 observation periods (baseline, P1, P2, P3).

• Evolution of the MCSMS score validated by ARIA and the European Academy of Allergy and Clinical Immunology (EAACI). The approach will be considered beneficial if a 30% reduction in this criterion is observed over the period D98-D112 compared to the baseline in patients with uncontrolled disease (VAS global allergy symptoms ≥20/100). However, the IRB requested that all patients should be included and this exclusion criterion has therefore been deleted.


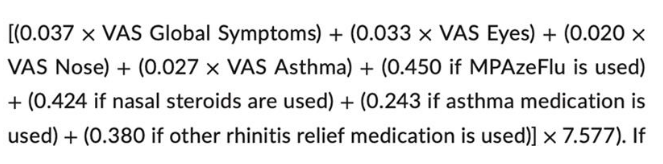


• Significant improvement of the individual’s discomfort in the eyes (VAS-eyes).

• Significant improvement of the individual's discomfort in the nose (VAS-nose).

• Significant improvement of the individual's discomfort in his/her work or studies (VAS-work, VAS-studies).

• Significant improvement of the "overall" VAS during the baseline observation periods, D42-D56 and D70-D84.

• Significant reduction in the proportion of days with uncontrolled AR (VAS overall discomfort >20) over the periods of symptom monitoring (Baseline, D42-D56, D70-D84, D98-D112).

• Evolution of the number of days during which the patient takes medicinal treatment over the periods of symptom follow-up (Baseline, D42-D56; D70-D84; D98-D112).

**Table S5 online: Detailed study protocol**

Cats’ food transition: A food transition period will be observed between D14 and D28, according to instructions provided by the Physician on D0.

The test food regimen was followed from D28 to D112:

- Cat(s) is/are fed with the test kibbles for 3 months (from D28 to D112)

D0-D112: MASK-air app

Patients will complete the daily questionnaires of the app during 4 periods of the study: [D0-D14], [D42-D56], [D70-D84] and [D98-D112].

Check compliance to food regimen

Inclusion visit (D0 - visit#1)

- Patient data (eCRF)
  - Demographic data and characteristics (M/F, age, allergy, etc.)
- Anonymised questionnaire: (ePRO)
  - Number of cats
  - Cat(s)’s body weight
  - Question about current methods to reduce cat allergen
  - Question about the relationship between the patient and the cat
- MASK-air:
  - Symptoms completed on MASK-air

From D0 to D14: Baseline data collection (MASK-air app)

- Symptoms

From D42 to D56 and from D70 to D84: Data collection during test food regimen (MASK-air app)

- Symptoms

D77: follow-up visit (visit #2)

- Questionnaire: (ePRO)
  - Question about current methods to reduce cat allergen
  - Question about the relationship between the patient and the cat
- Symptoms completed on MASK-air

From D98 to D112: Data collection during test food regimen (MASK-air app)

- Symptoms completed on MASK-air

D112: End of study visit (visit#3)

- Questionnaire: (ePRO)
  - Question about current methods to reduce cat allergen
  - Question about the relationship between the patient and the cat
- Symptoms completed on MASK-air

**Table S6 online: Instructions given to the patient by the physician at enrolment**

Questions will be asked by the allergist during the medical visits.

For the first questionnaire, the patients can tick as few or as many items that describe the methods they are using.

**What methods are you currently using in the home to manage cat allergens? (Select all that apply)**

- Having fewer cats in my home than I would like to have
- Washing my hands immediately after handling my cat(s)
- Selecting a cat breed that is reportedly less allergenic/hypoallergenic
- Restricting cat(s) to certain areas of house
- Avoiding or minimising direct contact with my cat(s) (e.g. holding my cat(s) or letting it/them sit on my lap)
- Excluding cat(s) from the bedroom
- Bathing my cat(s)
- Utilising HEPA filters/air purifier
- Utilising HEPA filters in vacuum cleaners
- Using covers on my furniture
- Removing/minimising carpeting in house
- Cleaning the home
- Other (Specify)
- None

For the second set of questions, the 1-7 scale applies to each of the 4 bulleted statements in that grouping.

**On a scale of 1-7, with 1=strongly disagree and 7=strongly agree, please indicate your agreement with each of the below statements.**

- I am able to have the relationship I want to have with my cat(s)
- I am able to interact physically with my cat(s) as much as I want to
- I am able to spend the amount of time I want to spend with my cat(s)
- I have to restrict my cat(s) from certain areas of my home (such as my bedroom)

**Table S7 online: Characteristics of the cats**

|  | Cat 1 | Cat 2 |
| --- | --- | --- |
| N patients | 44 | 15 |
| Age (years, mSD) | 2.1±0.5 | 1.9±0.6 |
| has lived in the household for more than 1 month (%) | 100 | 100 |
| is a female (%) | 55 | 40 |
| has short hair (%) | 75 | 67 |
| is sterilised (%) | 98 | 87 |
| lives mostly indoors (%) | 80 | 93 |

**Table S8 online: Interactions between patients and their cat(s) before the study**

|  | Mean | SD |
| --- | --- | --- |
| I am able to have the relationship I want to have with my cat(s) | 4.5 | 1.9 |
| I am able to interact physically with my cat(s) as much as I want to | 4.4 | 2.0 |
| I am able to spend the amount of time I want to spend with my cat(s) | 4.0 | 1.9 |
| I have to restrict my cat(s) from certain areas of my home (e.g. my bedroom) | 4.4 | 2.5 |

**Figure S1 online: Study flow**

*Baseline: D0-D14, P1: D42-D56 (start 2 weeks after the cat diet transition), P2: D70-D84 and P3: D98-D112*

**Figure S2 online: Number of MASK-air® days provided by study participants during (A) all the assessments and (B) each assessment period**

**
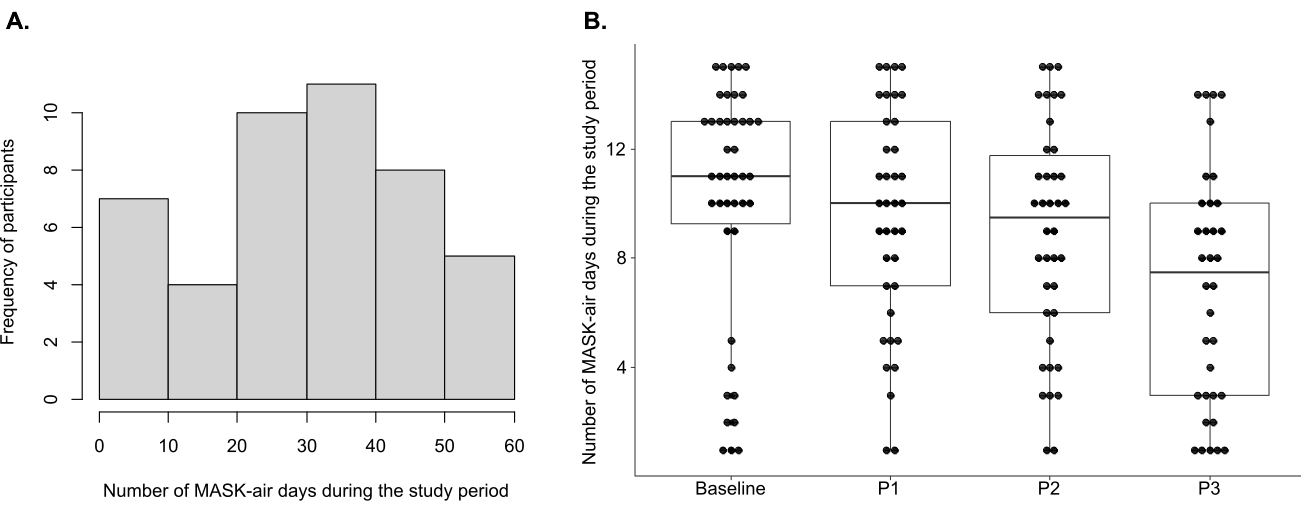
**

Baseline: Days 0-14, P1: Days 42-56, P2: Days 70-84, P3: Days 98-112

**Figure S3 online: Use of the app at the different evaluations**

**Figure S4 online: Individual data of the VAS scores and CSMS in 25 patients with all 3 evaluations**

**
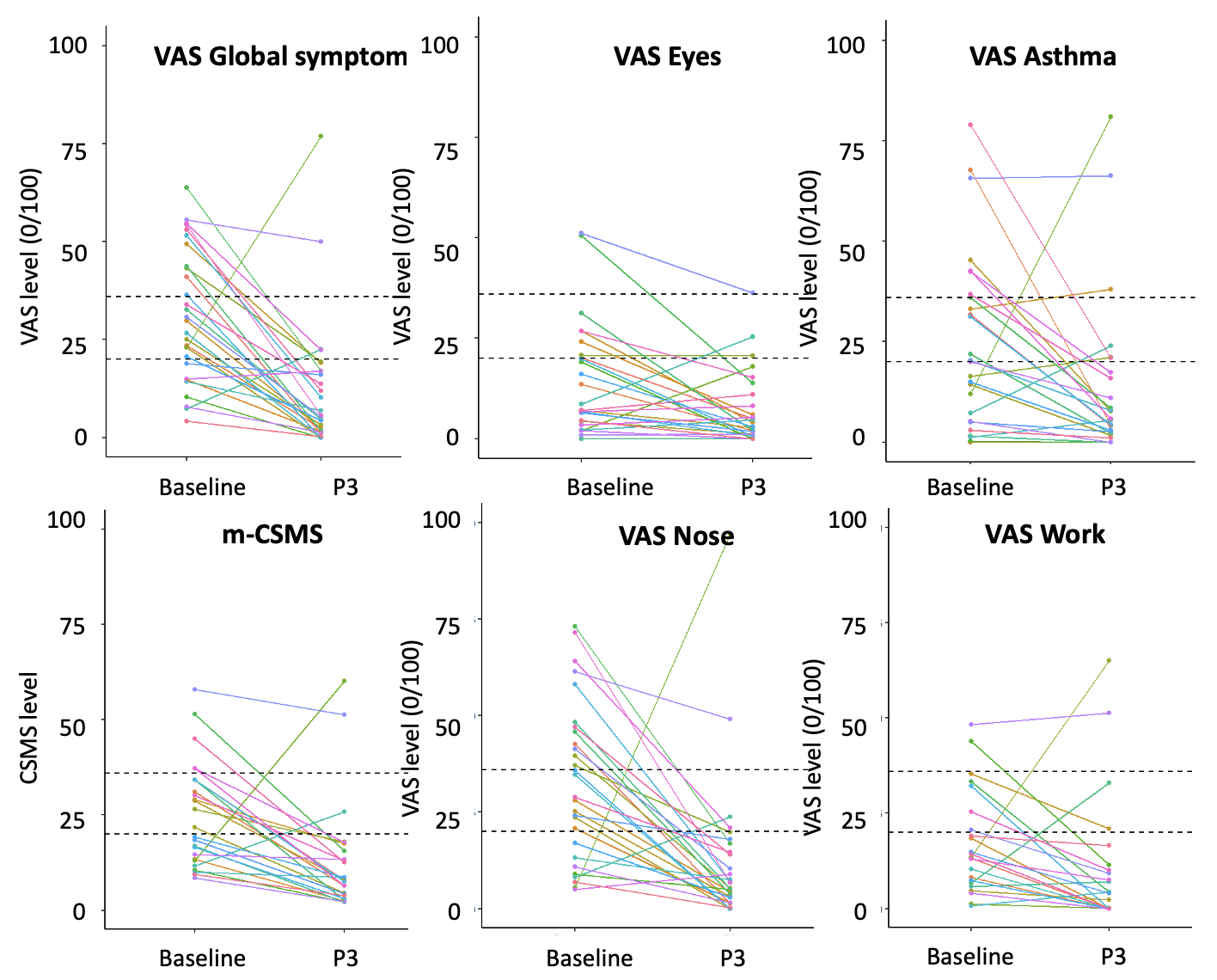
**
